# The palette of techniques for cell cycle analysis

**DOI:** 10.1002/1873-3468.13842

**Published:** 2020-05-22

**Authors:** Anna E. Eastman, Shangqin Guo

**Affiliations:** Department of Cell Biology and Yale Stem Cell Center, Yale University, New Haven, CT, USA

**Keywords:** cell cycle phases, cell cycle speed, heterogeneity

## Abstract

The cell division cycle is the generational period of cellular growth and propagation. Cell cycle progression needs to be highly regulated to preserve genomic fidelity while increasing cell number. In multicellular organisms, the cell cycle must also coordinate with cell fate specification during development and tissue homeostasis. Altered cell cycle dynamics play a central role also in a number of pathophysiological processes. Thus, extensive effort has been made to define the biochemical machineries that execute the cell cycle and their regulation, as well as implementing more sensitive and accurate cell cycle measurements. Here, we review the available techniques for cell cycle analysis, revisiting the assumptions behind conventional population-based measurements and discussing new tools to better address cell cycle heterogeneity in the single-cell era. We weigh the strengths, weaknesses, and trade-offs of methods designed to measure temporal aspects of the cell cycle. Finally, we discuss emerging techniques for capturing cell cycle speed at single-cell resolution in live animals.

Eukaryotic cells progress through sequential molecular events to complete a round of the mitotic cell cycle: gap phase 1 (G1), DNA synthesis (S), gap phase 2 (G2), and mitosis (M), with interphase comprising all cell cycle phases but mitosis [1,2]. Cells can exit the cell cycle after mitosis and enter into G0 [3], a reversible state of cell cycle arrest referred to as quiescence. Cell cycle arrest at other phases is typically symptomatic of stress or excessive damage to DNA or various cellular machineries [4], although it can also be the result of active regulation, exemplified by G2 quiescence in adult neural stem cells [5]. Cells that are metabolically active but cannot divide, due to shortened telomeres and/or accumulated DNA damage, are termed senescent [6], while postmitotic cells are those that have exited the cell cycle upon terminal differentiation, such as neurons [7]. Of note, these noncycling state are distinct: Senescent cells cannot re-enter the cell cycle [6], while postmitotic cells can [8], demonstrating that cell identity specification suppresses replicative capacity rather than causing its permanent loss.

Measuring the cell cycle can include probing many aspects: whether or not a cell is cycling or whether it retains the potential to cycle; which cell cycle phase a cell is in, or whether it has progressed beyond a given point of interest, such as a checkpoint or the completion of DNA replication; the dwell-time in a particular phase; the number of mitoses completed by a cellular lineage in a certain timeframe; or the total duration of the cell cycle. Any of these parameters could be used to describe populations or individual cells. Features with a time component can be deduced retrospectively after completion of a given cell cycle-related process, or in real time while it is taking place, focusing on a single time point or through continuous sampling. We review existing and emerging techniques for measuring the cell cycle along with their relative benefits, limitations, and trade-offs. Like paints on a palette, these techniques offer multiple choices for portraying cell cycle states either in quick, minimal strokes, or in laborious, exquisite detail. While these techniques are sometimes interchangeable options providing similar results, more often one is clearly the only appropriate choice to fulfill a given need. Proper choice of complementary techniques should paint the most accurate and vivid picture of this dynamic biological process.

## Biochemical definition of the eukaryotic cell cycle machinery: a historical perspective

Cell cycle progression is fueled by a series of orderly protein phosphorylation, degradation, and new expression events, a conserved theme extending from yeast to humans. Yeast genetic screens identified major cell cycle regulators, such as cell division cycle 2 (CDC2), which was found to be required for G2/M and G1/S transitions [9]. Further work revealed CDC2 to be a kinase, whose activity is controlled by other cell cycle regulators, including by WEE1-mediated phosphorylation and inactivation in G2 phase [9]. Its cell cycle promoting activity depends on the concentration of its cyclin binding partners, the levels of which rise and fall throughout the cell cycle. Identical modes of regulation by CDC2 homologs were demonstrated in frogs and other vertebrates, notably humans, whose CDC2 homolog is named cyclin-dependent kinase 1 (CDK1) [9]. Classical biochemical delineation of the cell cycle benefitted from several unique features of amphibian eggs: They can be harvested relatively easily, manipulated *ex vivo*, contain large quantities of cytoplasm, and their maturation through the mitotic cell cycle can be conveniently controlled [10]. Dynamical modeling synthesized the actions and interactions of individual cell cycle molecular players into an elegant paradigm of a biological oscillator, propelled forward by threshold-sensitive positive-feedback switches and continuous delayed negative feedback regulation [11,12]. CDK1 in G2, for example, positively regulates the anaphase-promoting complex (APC), the E3 ubiquitin ligase that drives transition into mitosis via proteolytic destruction of various cell cycle regulators, including CDK1 itself [12]. When CDK1 expression is activated again in late G1, its phosphorylation targets include transcriptional repressors that shut down G1 gene expression to drive S-phase commitment [13].

## Cell cycle as a major source of cellular heterogeneity unveiled in the single-cell era

Cell cycle progression is regulated by mitogens and cell cycle inhibitors that collectively govern when, whether, and how frequently division takes place. Transformed cells often break loose from these constraints via gainof-function mutations in mitogenic regulators like components of growth factor signaling networks, or loss-of-function mutations in the cell cycle’s braking system, including CDK inhibitors and DNA damage response factors [14,15]. As cancer involves overproliferation, the cell cycle has been a major target of anticancer drugs [14]. Not surprisingly, however, cell cycle-targeting drugs often suffer limited specificity and/or frequent drug resistance [16].

Pre-existing cell cycle heterogeneity at the time of treatment is one determinant of outcome. Drugs aimed to kill proliferative cells may spare quiescent cancer stem cells, leaving them to re-seed the cancer after therapy [17]. Stimulating the proliferation of cancer stem cells increased their vulnerability toward cell cycle chemotherapy in animal models of acute myeloid leukemia [18]. However, in order to fully understand what defenses and/or vulnerabilities define cancer stem cells in nonhematopoietic malignancies, methods for identifying them are needed. Universal markers for cancer stem cells across multiple tissues remain elusive, leading to the use of cell cycle behavior as an identifying feature [19]. Since chemoresistance is believed to be enriched among slow- or noncycling cells, sensitive sorting methods based on cell cycle kinetics are promising.

The complex- and context-dependent effects of cell cycle inhibitors [20], targeting CDK4/6 and others, continue to be uncovered, adding to the list of novel, cell cycle-independent functions of cyclins and CDKs [21,22]. Moving into the age of personalized medicine, high-throughput drug screening of individual patient samples coupled with omics analyses can reveal links between cancer genotype and sensitivity/resistance to specific drugs, including cell cycle modulators [23]. The question then becomes not why, but how to refine the search to pinpoint susceptibilities that might occur only in specific cells at specific times. This is a problem requiring tools for dissecting cell cycle heterogeneity.

## Synchronization strategies for dissecting cell cycle control

### Chemical synchronization

Since most mammalian cells divide asynchronously, forcing them to accumulate in a particular cell cycle phase can be advantageous for assays requiring large quantities of sample. There are many chemical synchronization protocols targeting different phases [24]. For example, the cholesterol synthesis inhibitor lovastatin arrests cells in G1 [25]; the DNA polymerase inhibitor aphidicolin halts S-phase progression [26], and the microtubule polymerization inhibitor nocodazole disrupts the mitotic spindle to block cells in M-phase [27]. The efficiency of synchronization varies by drug, and the dose/duration of the chemical block must be optimized for every cell type. Increasing dose/duration can improve purity by reducing the number of cells that escape the block, at the expense of viability. It is widely acknowledged that even temporary cell cycle arrest could be cytotoxic, resulting in altered cellular behavior such as increased apoptosis. This concern is especially relevant for studies of cell cycle in the context of rare fate transitions, where minor perturbations to cell growth and viability may have an outsized impact on fate outcome.

### Drug-free synchronization

The cell cycle can be synchronized without the use of chemicals. Commonly used methods include serum/growth factor starvation [26,28], contact inhibition [28], mitotic shake-off [29], and size-based elutriation [30,31]. Serum starvation and contact inhibition cause cells to arrest in G0, as cell cycle entry requires nutrient and space availability [26,28]. However, like druginduced arrest, synchronization via starvation is not 100% efficient and can introduce stress artifacts [26]. Serum starvation and contact inhibition are ineffective for many cancer cell lines, whose proliferation is less sensitive to metabolic and crowding-mediated arrest [25]. Mitotic shake-off is a procedure that can increase purity of cell cycle synchronization while salvaging viability. Since adherent cultured cells temporarily lose most contact with their substrate during mitosis/cytokinesis, they can be freed through mechanical agitation and then collected in the supernatant. Mitotic shake-off is typically done in combination with mitosis-arresting drugs, but can also be performed on asynchronous cells [29]. The yield from the latter method would likely be lower by 1–2 orders of magnitude, given that M-phase cells are a minor fraction of asynchronous cell populations. Centrifugal elutriation fractionates cells based on their size, which in principle correlates with cell cycle progression [32]. Therefore, this method is only applicable to cell populations that are sufficiently homogeneous in size [30].

### *In silico* synchronization

Single-cell data acquired from physically asynchronous samples can be synchronized during data analysis. *In silico* synchronization can be used in multiparameter assays such as flow cytometry and microscopy: Cell cycle phase is determined and ordered sequentially based on cell cycle phase markers, and then, other experimentally measured features are analyzed as a function of cell cycle phase. The number of parameters can be increased to thousands in single-cell mRNA sequencing (scRNA-seq), followed by bioinformatics processing to reconstruct cell cycle phase from gene expression data [33].

Assigning cell cycle phase in genomic analysis can reduce noise, as differences in cell cycle and related genes often dominate scRNA-seq data. It is not uncommon to first discard annotated cell cycle genes whose oscillations would otherwise overwhelm and mask ‘true’ biological differences. However, the cell cycle can be so interconnected with cellular states that even genes not strictly classified as ‘cell cycle’ nevertheless correlate with at least one cell cycle gene [34]. Simply throwing away cell cycle genes may not be optimal when the annotated gene lists do not sufficiently account for the cell cycle-driven heterogeneity, or when cell cycle genes are important beyond their immediate role in cell cycle progression. Therefore, bioinformatics workflows involving *a priori* cell cycle phase assignment could be advantageous [34].

## Methods to determine cell cycle phases

### Probes and labels

The presence of cycling activity can be determined by DNA-binding dyes on the basis of ploidy, since cells double their genomic content during S-phase [35,36]. Fluorescent analysis of a stained population of cells produces a DNA histogram: Diploid cells (2N) in G0/G1 have half the amount of DNA as G2 cells (4N), while S-phase cells in the process of genome replication have an intermediate DNA amount. 4’,6-diamidino-2-phenylindole (DAPI) and propidium iodide (PI) are commonly used to stain fixed cells, while other DNA dyes such as the Hoechst and DRAQ families are suitable for live-cell analysis [37]. RNA-binding dyes such as pyronin Y (PY) can further separate 2N cells into G1 or G0, since low transcriptional activity is part of the metabolic dormancy associated with G0 [35]. The mitotic chromosome-dispersing protein Ki-67 [38] is another marker of actively cycling cells, as are the family of replication licensing proteins, Mcm2–7 [39].

Cells actively engaged in DNA replication can be identified using thymidine analogs, which are incorporated into nascent DNA during the labeling period [35]. This family of probes share a similar mechanism of action, but their labeling/detection protocols carry distinct advantages and disadvantages [40]. Halogenated thymidine nucleosides like BrdU, CldU, and IdU are detected by immunostaining following fixation, permeabilization, and DNA denaturation. Despite the multiple steps in the staining protocol, these molecules, particularly BrdU, largely replaced tritiated thymidine (H^3^-thymidine) in common use since antibody labeling circumvents the need for radioisotopes. An alkylated thymidine nucleoside, EdU, can be detected by copper-catalyzed ‘click’ chemistry [41], which has the advantages of superior labeling sensitivity and minimal disruption to the DNA staining signal as no DNA denaturation is required [40,41].

Phosphorylated histone H3 (pH3), present from late G2 through telophase [42], can be recognized by specific antibodies [35,42] and is used as a marker of M-phase cells. The frequency of mitotic cells in an immunocytochemical assay is known as the mitotic index [43] and is used to compare proliferative rates between populations or pathological states.

Accurate cell cycle profiling requires the exclusion of dead/dying cells from the analysis, as they do not conform to cell cycle-regulated differences in nucleotide metabolism and chromosome configuration. This can be accomplished using stains that cross the leaky membranes of dead/dying cells to permanently and nonspecifically bind proteins, thereby discriminating live/dead cells in a manner that can withstand fixation [44]. DNA dyes such as 7-AAD and PI can similarly identify dead/dying cells in unfixed samples [45]. Other common apoptosis markers include Annexin V and terminal deoxynucleotidyl transferase (TdT) dUTP nick-end labeling (TUNEL) positivity [45].

Contrasting the detection of cell cycle phases, universal identifiers of cellular senescence are lacking. Commonly accepted senescence markers include increased β-galactosidase or the cell cycle inhibitors p16^Ink4A^ and p21 [46]. Additionally, the International Cell Senescence Association (ICSA) has recently published a standardized workflow for detecting cellular senescence and evaluating subcategories of senescence-associated phenotypes, such as high extracellular secretion, extensive DNA damage, and oxidative stress [46].

A host of well-characterized cell cycle genes uniquely expressed within specific cell cycle phases provides endogenous targets for antibody-based classification [35,47]. Because these are mostly found in the nucleus, such staining assays require fixation and permeabilization. Common methods for cell cycle phase determination are summarized in Table 1.

### Live cell cycle reporters

Fluorescent protein tags circumvent the need for fixation and allow key moments during the cell cycle to be identified on the basis of morphology [48–50], subcellular localization [50–52], and/or intensity. Examples of morphological probes include fluorescent proteintagged histones, which report mitotic progression based on characteristic chromatin condensation and alignment [48]. Fluorescently tagged PCNA coalesces into replication foci during S-phase [49]. Tagging proteins that undergo cell cycle-regulated nuclear translocation, such as human DNA helicase B [50] and cyclin B1 [51], yields nuclear localized fluorescence in specific cell cycle phases. The FUCCI family of reporters harnesses the proteolytic nature of the cell cycle machinery, such that the expression of each fluorescent probe is restricted to a specific cell cycle phase [53,54]. The original FUCCI is a two-color system with distinct fluorescent proteins fused to CDT1 and GEMININ, marking G0/G1 and S/G2, respectively [53]. A clear benefit of discriminating cell cycle phases by fluorescence intensity is that it allows cell separation by fluorescence activated cell sorting (FACS). A more recent version adds a third tagged protein, SLBP, to distinguish S-phase from G2, and a fourth reporter fused to histone H1.0 to identify early M-phase cells based on chromatin condensation in microscopy-based assays [54]. As the original CDT1 reporter signal was found to persist beyond S-phase entry, an alternative reporter, PIP-FUCCI, was developed to improve temporal precision [55]. This system uses a replicationcoupled degron that is sensitive to the presence of DNA-bound PCNA to restrict a fluorescent protein from being expressed during S-phase [55]. Some cell cycle reporters, such as fluorescently tagged CDT1 [56] and PCNA [57], show signal accumulation over time within the reported phase, facilitating cell cycle phase subdivision into early vs. late stages based on signal intensity when transgene copy number/genomic integration is controlled. As long as there are enough visually identifiable features demarcating cell cycle phase progression, these reporters can be used singly [57] or combinatorially [50,54,58] in time-lapse microscopy to quantify cell cycle phase duration at the single-cell level. The approaches highlighted above, and other commonly used cell cycle markers, are summarized in Table 1.

### Classification by artificial intelligence

To reduce the number of fluorescent probes or to study cells in their native, undisturbed form, label-free techniques to infer cell cycle phases from asynchronous populations have been developed [59,60]. Advances in high-speed optical capture and digitization technology combined with postacquisition reconstruction based on deep learning allow high-throughput measurement of many cellular features—including those associated with the cell cycle—from microscopy [61–64] and image flow cytometry [60,65,66]. Artificial intelligence (AI) is a powerful tool for unlocking hidden quantitative features from complex optical data. The next decade will likely embrace AI as the workflows become more user-friendly.

## Assessing cell cycle dynamics

While there are many ways to directly evaluate cell cycle phase, dynamical features such as cell cycle duration—the time interval between corresponding phases of consecutive cell cycles—can be more difficult to determine when time-lapse microscopy is prohibited. The length of this interval, also referred to as cell cycle speed, is increasingly recognized to be significant for many cell fate decisions [67–73]. When cells can be imaged and tracked longitudinally, cell cycle speed is most easily measured based on the interval between two consecutive mitoses, as cell division is the most morphologically recognizable event in the cell cycle. A related but distinct metric is the duration of specific phases. A range of experimental techniques offer varying degrees of sensitivity and accuracy, with some affording single-cell resolution.

One of the oldest and most basic approaches for evaluating cell cycle duration is to look for changes in the overall population size. Counting the number of cells over a time course measures the rate of a population’s expansion, or population doubling time, which is sum of proliferation and death. However, while population-level behaviors may reflect underlying differences in cellular dynamics, population readouts alone cannot reveal what the differences are nor which cells are affected. For example, when a population shows mild increase after a particular treatment, it could come from modest cell cycle acceleration in a large number of cells; dramatic acceleration in a small number of cells; dramatic acceleration in a large number of cells with nearly equivalent increase in apoptosis; or anything in between. Importantly—especially for clinically relevant problems like cancer drug efficacy—multiple different responses could be occurring at the same time in distinct cellular subpopulations. With this in mind, we will discuss common and emerging techniques for the analysis of cell cycle dynamics. Their relative advantages and disadvantages are discussed below and summarized in Table 2.

### Inferring cell cycle progression from snapshot data: the ergodicity assumption

**Fig. 1.**
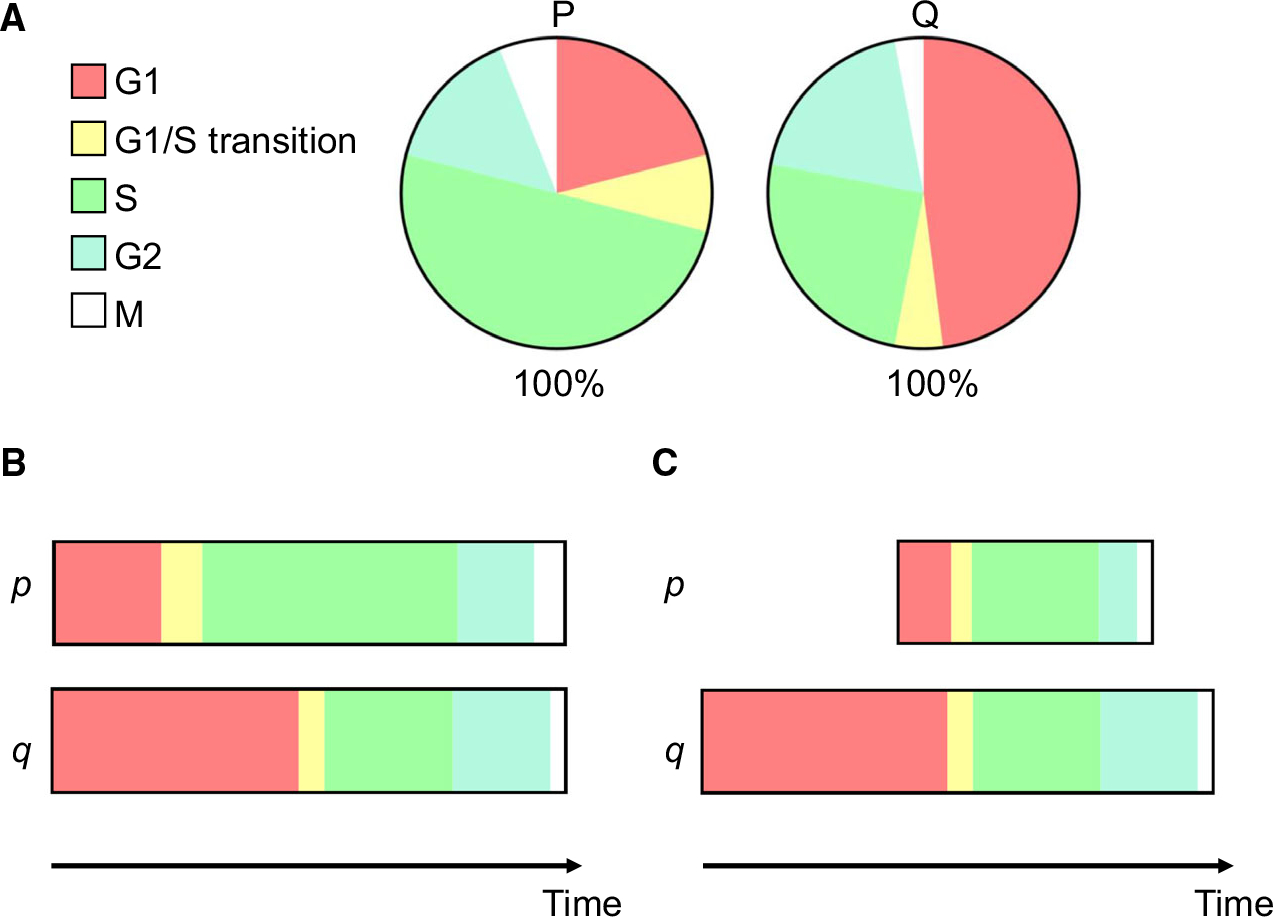
Interpreting dynamics from cell cycle phase distribution with ergodic assumptions. (A) Pie charts showing the percentage of cells identified in each phase of the cell cycle at a single moment in time in two populations, P and Q. When P and Q’s individual cellular constituents, *p* and *q*, cycle asynchronously with homogenous kinetics, the cell cycle phase distribution of P and Q reflects the relative duration of each phase within the total interval of a single cell cycle. (B) If P and Q have equivalent doubling times, then differences in *p* and *q*’s transit time through each phase can be imputed from differences in population fractions as in (A). (C) Alternatively, if the duration of a particular phase (e.g., S) is known to be equivalent in P and Q, then (A) infers differences in the total cell cycle length of *p* and *q*.

Cell cycle phase markers such as those listed in Table 1 give a binary answer (Yes/No): Is this cell in *X* phase? In synchronized cells, phase markers indicate the efficiency of synchronization; followed through a time course, they reflect cell cycle progression into or out of a particular phase. In asynchronous cells, cell cycle phase markers are often used to compare the proliferation kinetics. This approach rests on the ergodic principle as applied to cell cycle, which assumes that within asynchronous populations, the fraction of cells in a given phase at a single timepoint equals the proportion of time a single cell spends in that phase relative to the total cell cycle duration [74,75] (Figure 1). Therefore, cell cycle phase lengthening or shortening are inferred by differences in the frequency of cells in a particular phase.

DNA content histograms are classically used in this way, with a change in the relative size of the 2N peak (encompassing cells in G0/G1) interpreted as altered dwell time in G0/G1 relative to S/G2/M. Since slow cellular turnover is most often associated with a long G0/G1 dwell time, DNA histograms with prominent 2N peaks and few > 2N cells are taken as evidence of a slow or mostly dormant cell cycle. Other common labels using similar principles are S- and M-phasespecific markers such as BrdU and pH3. Although the length of any cell cycle phase can vary—particularly in response to DNA damage [76]—variation in total cell cycle time predominantly results from variable G0/G1 [77] and/or G2 phase length. This can be a handy simplification when transit times through mitosis and S-phase are comparatively constant, allowing experimentally observed differences in M- or S-phase labeling frequency at the populational level to indicate differences in overall cell cycle time. M-phase in particular is considered invariable in length [78], although biologically intriguing exceptions do exist. For example, in the developing mouse neocortex, prolonged mitosis in radial glia is associated with divergent fate commitment [79].

Caution must be used when inferring cell cycle rate differences from a single S-phase label (BrdU or similar), since the rate of DNA replication (and consequently the duration needed to complete S-phase) can change concomitantly with fate specification [70,80]. Indeed, the overall cell cycle accelerates by S-phase shortening during erythroid terminal differentiation [70] and neocortical neural progenitor differentiation [80]. In these circumstances, data from a single S-phase label would be misleading because a shortened S-phase would reduce the fraction of BrdU+ cells without implying a longer cell cycle [81]. A useful parameter in diagnosing this possibility is the median BrdU intensity among the S-phase cells. BrdU median fluorescent intensity, or MFI, reflects the amount of BrdU incorporation and hence the rate of DNA synthesis during the pulse period [70]. A higher MFI suggests faster progression through S-phase. In fact, one method exploiting the superior detection sensitivity of EdU [40] derives the length of S-phase based on the shortest labeling time required to achieve maximum cellular EdU intensity, which is expected to occur in cells exposed to the label throughout the entirety of DNA replication [82]. A potential caveat of this approach is that the results might be biased toward cells with the shortest S-phase.

Cumulative labeling with a single probe [83] or sequential labeling [84] with multiple thymidine analogs calculates the average (mean) length of S-phase as well as the mean overall cell cycle time. In the sequential thymidine assay, cells are pulsed consecutively with two nucleosides just before fixation. The two nucleosides, such as EdU and BrdU, can be detectible separately. With the time interval between the addition of the first and the second label known, S-phase duration and overall cell cycle length are calculated from the fraction of cells which incorporated one or both of the labels [84]. This assay can be used to quantify mean cell cycle length in cultured cells as well as live animals [70,85]. As with all readouts that report a population average, the sequential thymidine assay is more accurate for samples with relatively homogeneous cycling kinetics.

While methods that invoke the ergodic principle can be time-saving and informative, the underlying assumptions should be validated. The accuracy of the readout also depends on homogeneous cell cycle dynamics. As cell cycle heterogeneity is prevalent [86], it is critical to recognize that the fraction of cells found within a specific cell cycle phase reflects the average population kinetics, but not necessarily individual cells within that population [75]. On the other hand, one reason such methods are in widespread use is that they allow glimpses of cell cycle dynamics when only a single, fixed timepoint is available. This most notably applies to primary cells/tissues derived from humans, which are simultaneously the most clinically relevant and also the least accessible and experimentally tractable.

### Label-chase approaches

Cell cycle dynamics measured using any of the abovedescribed techniques reflect a population average. Other approaches are needed to unmask cell cycle speed heterogeneity, especially when cells with distinct proliferative behaviors are to be isolated by cell sorting. Label retention assays exploit the dilutional nature of cell divisions to estimate how many mitoses a cell experienced since being marked with a fluorescent label. Labels can be in the form of chemical dyes [87] or experimentally expressed fluorescent molecules [88–90]. The salient requirement is that it cannot be acquired anew but can only be lost through successive rounds of cell division. The degree of weakening in cellular fluorescent intensity reflects the number of divisions undergone during the chase period, creating an opportunity for cell sorting by FACS.

Fluorescent chemical dyes are well suited to experimental systems with relatively synchronous divisions and homogeneous cell size, such as activated lymphocytes [91]. However, many cell types do not form well-resolved dye intensity peaks due to their inherent heterogeneity at the time of labeling [92]. Furthermore, the dyes have a certain level of cytotoxicity [93], which must be considered if the goal is to sort live cells for continued functional analyses. Adherent cells must be enzymatically dissociated into single-cell suspensions before labeling, which can introduce additional artifacts. The optimal length of the chase period is dictated by the target cells’ typical cycling rate. It is important to note that the dye dilution assay is a historical measurement based on the number of divisions over the chase period, not necessarily the cell cycle speed at the time of measuring. Finally, the sensitivity of the dye dilution assay could be limited in populations where differences in cycling rate are subtle [94].

Similar sensitivity limitations apply to the equivalent *in vivo* technique, which uses transgenic H2B-GFP under the control of an inducible promoter to label chromatin. For example, the Tet-Off H2B-GFP system excels at identifying very slow- or nondividing cells, such as dormant tissue stem cells [88,89], which retain bright GFP signal months after transgene expression is switched off. Conversely, short chase periods (24–48 h) are required to capture the fastest cells before the label falls below the detection limit in most cells. Because they are compatible with FACS, label retention assays offer the distinct advantage of enabling downstream functional comparisons between populations with different cycling kinetics—an essential tool for dissecting the role of proliferation rate in cell fate decision making.

### Capturing cell cycle dynamics through live-cell imaging

The most direct way to measure cell cycle speed is to capture consecutive mitoses using time-lapse microscopy. Embryonic cell cycle dynamics have been captured in this manner for a variety of species [95–99], including human embryos with direct applications for reproductive medicine [100]. *In vitro* fertilization (IVF) clinics have used time-lapse imaging to select, among the laboratory-generated early embryos, which are the most likely to implant and develop. Cell cycle length in the first four embryonic divisions was found to be a critical predictor of successful development [100]. The narrow cell division timing differences between embryos which are unlikely to gestate compared to those that may one day walk prove yet again that cell cycle length is fundamentally important and needs to be critically regulated.

Technological advances in light microscopy have greatly improved our ability to capture single-cell behavior in thick specimens without overt phototoxicity. Light-sheet microscopy is a popular technique for embryo live imaging owing to its excellent spatial and temporal resolution with less photodamage than confocal microscopy [101,102]. Multiphoton microscopy allows high-resolution intravital live-cell imaging [103] in dense, three-dimensional tissues such as mouse hair follicles [104], intestine [105], and brain [106]. Surgically implanted optical windows or probes allow repeated imaging of the same location [107], although the duration of a single imaging session is limited by the need to restrain and sedate animals. Tracking cycling behavior of single cells *in vivo* is therefore limited to cells that divide slowly enough to be captured with sparse time intervals, but fast enough to take place within a maximum of 40 h under the most carefully controlled anesthesia [108]. The advantages of time-lapse microscopy are single-cell precision and ground-truth accuracy, yet the dynamical heterogeneity revealed by this method is retrospective. When physical segregation of cells with distinct cycling rates is necessary for comparative downstream analysis, microscopes equipped with robotic picking devices [109] can be used to pluck cells of interest after their identification by time-lapse imaging.

A predictive modeling tool harnessing freely available single-cell time course data provides another alternative: the virtual experiment [110]. Unique among the tools discussed so far, the Cell Cycle Browser [110] provides users with the experimental workflow to explore potential relationships between common cell cycle parameters and/or regulatory proteins. Modeling and simulation tools allow users to generate predictions about the consequences of introducing perturbations.

High-throughput image analysis is often labor-intensive, computationally expensive, and requires customized solutions [64,111]. Developing computational tools to partially or fully automate quantitative data extraction from micrographs is its own discipline. In the age of AI, automated software tools for image processing and analysis [64,112–115], including cell tracking [116], are becoming more versatile. Fully automated cell tracking is a laudable goal, but for behaviors occurring on timescales of days to weeks (such as sequential cell cycles), even small errors can lead to invalid data [111]. For this reason, ironically, the recent innovation in automated cell tracking has been to improve user manual control to correct cell tracking errors [111]. The AI-based image analysis workflows, including those for longitudinal cell tracking and quantitative feature analysis, will become more widely adopted with improved user interfaces [117].

### Resolving live cell cycle speed with a single snapshot: a reporter that captures the heterogeneous landscape of cell proliferation *in vivo*

**Fig. 2.**
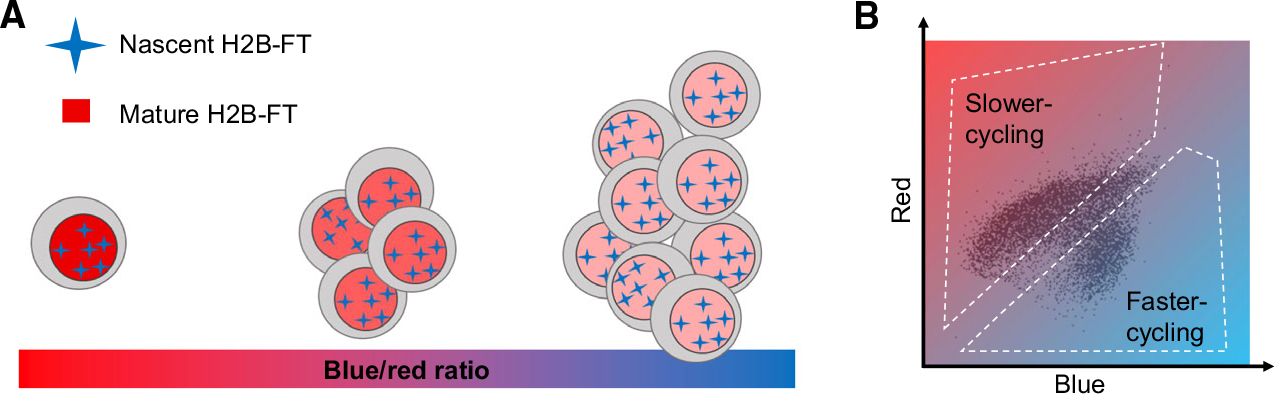
H2B-FT reports live cell cycle speed heterogeneity in snapshot measurements. (A) H2B tagged with a color-changing fluorescent timer (FT) protein reports cell cycle speed heterogeneity due to the divergent half-lives of the nascent (blue) form as compared to the mature (red) form. While mitosis halves cellular levels of both the blue and red species, the red form’s greater stability allows it to accumulate while the short-lived blue form cannot, making red levels sensitive to modulation by cell division frequency. Faster-cycling cells are therefore distinguishable from slower- or nondividing cells by their higher intranuclear blue/red fluorescence ratio. (B) Cells with distinct proliferation rates can be identified in a two-color fluorescence assay, such as flow cytometry.

The fundamental importance of proliferation control in development, regeneration, and disease necessitates the measurement of cell cycle speed besides describing the specific cell cycle phases. Cell cycle speed has been challenging to determine in dynamic and heterogeneous systems where cells transition asynchronously through complex fate choices, especially *in vivo*. A novel reporter has been developed which combines the ease of snapshot data acquisition with time-resolved single-cell cycling dynamics [118]. The reporter, which consists of a color-changing fluorescent timer (FT) protein [119] fused onto a core histone, reports live cell cycle speed in a ratiometric readout [118] (Figure 2). The reporter takes advantage of the distinct half-life of the two FT fluorescent species [119] and their different susceptibility to dilution by cell division [120]. The long-lived species (red) accumulates steadily in slow-dividing cells, but less so in fast-dividing ones, whereas the blue species is too short-lived for its concentration to be affected by cell cycle length. The blue/red ratio is therefore related to cell cycle length, with shorter (faster) cell cycles exhibiting a high blue/red ratio (Figure 2). The H2B-FT reporter ranks cells based on their proliferation rate in a single snapshot measurement, making it appealing for use in live animals as long as the blue/red fluorescence can be determined from microscopy or flow cytometry, with the latter providing the means also for live cell sorting. The resolution of the current H2B-FT reporter is better-suited for faster-cycling mammalian cells; quantitative estimates of cell cycle length below ~ 30 h per cycle can be generated. Its resolution in slower-dividing cells becomes limited. In this respect, it is the opposite of the Tet-Off-H2B-GFP label retention reporter, in which the extremely slow or nondividing cells remain visible after several months of chase [88,89].

## Conclusion and future outlook

The power of unpacking cellular heterogeneity is ever more appreciated as single-cell technologies improve and evolve, revealing a dominance of cell cycle in driving cellular heterogeneity. Whether and how such heterogeneity dictates biology is still being investigated, but it is likely profound and mechanistically coupled to cell cycle dynamics [68]. Given the importance of cell cycle dynamics in various biological contexts, including cancer, innovative techniques continue to be developed to provide ever more accurate and better-resolved cell cycle information.

No single approach is without trade-offs. Understanding the limitations and assumptions underlying each method not only provides helpful guidelines in choosing the appropriate assay(s) for the question at hand, but also directs their further refinement. For instance, the H2B-FT reporter’s accuracy in quantifying cell cycle length [118] would likely be improved by pre-assigning cell cycle phases with a G1 or S/G2 reporter, given that some inherent noise emerges from the evolution of the blue/red ratio with time across a single cell cycle. Furthermore, a mutational variant of the FT with slower blue-to-red color change kinetics [119] is likely to extend its resolution range to slower-dividing cells. Snapshot ratiometric values derived for single cells have been used to report changes in cellular identity by the RNA velocity method [121]. As cell identity changes more readily in rapidly dividing cells [72,94,122,123], combining both ratiometric methods could offer unique insights into the regulation of cell fate by cell cycle dynamics. For microscopy-based methods, the ability to capture dividing cellular lineages in freely moving animals rather than those under prolonged sedation would be advantageous. Adapting the existing wearable technology for continuous, multiday intravital imaging could be anticipated [124]. New imaging or detection modalities would also require unprecedented ability to capture, process, analyze, and share the high-dimensional data, for which computational algorithms and artificial intelligence are expected to become more flexible, reliable, and accessible [113,117]. It is through the complimentary application of multiple techniques that the many facets of the dynamic cell cycle can be understood.

## Figures and Tables

**Table 1.** Common cell cycle markers.

Category	Common markers or reagents

DNA-binding dyes	4′,6-diamidino-2-phenylindole (DAPI), Propidium Iodide (PI), 7-Aminoactinomycin D (7-AAD), Hoechst, DRAQ5™
Proliferation vs. quiescence	Ki67, Mcm2-7, Pyronin Y, p27K
Dead/apoptotic markers	7-AAD, PI, Terminal deoxynucleotidyl transferase (TdT) dUTP Nick-End Labeling (TUNEL), Annexin V, LIVE/DEAD™ stains
Senescence markers	p21, p16^Ink4A^, Senescence-Associated β-Galactosidase (SAβG)
Nascent DNA labels	BrdU, IdU, CldU, EdU, 3H-Thymidine
G1 phase	CDT1, HDHB (nuclear)
S-phase	SLBP; Formation of PCNA foci; Geminin (present through S-G2)
G2 phase	Cyclin B1 (cytoplasmic); Geminin
M-phase	Phosphorylated Histone H3 (pH3); Cyclin B1 (nuclear); Condensed chromatin as detected by DNA-binding dyes or core histone antibodies

**Table 2.** A comparison of common and emerging techniques to study cell cycle dynamics.

Method	Use/Readout	Advantages	Limitations

DNA content dye	Snapshot of cell cycle phase distribution	• Rapid protocol • 1-time measurement • Live-cell dyes available • Enables FACS sorting	• Population level • Not a quantitative measurement of cell cycle phase duration
pH3 or single thymidine pulse	Frequency of cells in M-phase (pH3) or S-phase (thymidine analog)	• Robust, specific labeling • 1-time measurement • Acceptable for qualitatively comparing proliferation rates between populations^a^	• Population level • Not a quantitative measurement of cell cycle phase duration • Requires fixation
Cell counting time course	Measures population growth over time	• Straightforward protocol • Reliable quantification of doubling time (when combined with an assay to measure cell death rate)	• Impractical or currently not possible *in vivo*^b^ • Doubling time represents population average • Multiple measurements
Live cell cycle phase reporter	Monitors cellular status with respect to a particular cell cycle phase	• Live cell assay • Continuous monitor • Single-cell resolution (when combined with time-lapse microscopy) • Most reporters enable FACS	• Single snapshot yields limited inferences about cell cycle dynamics (similar to DNA dyes or fixed-cell cycle markers)
Time-lapse microscopy	Direct visualization of live cells over time	• Definitive, ground-truth measurement of cell cycle rates • Single-cell resolution • Spatial information retained • Allows assessment of other cellular features, for example, morphology and migration	• Large data files • Time-intensive postacquisition processing/analysis • Single-cell tracking could be difficult for some cell types • Challenging or currently limited *in vivo*^b^ • Information gained is retrospective • Conditions require careful optimization (e.g., temperature, gas, phototoxicity, evaporation)
2-Thymidine assay	Quantification of S-phase duration and cell cycle period	• Single snapshot • Allows cell cycle length quantification in otherwise inaccessible tissues	• Difficult protocol requiring many starting cells • Population average • Validity depends on population homogeneity • Requires fixation
Fluorescent dye retention assay	Identification of cells which have undergone one or more divisions based on dilution of a fluorescent dye label	• Enables FACS-based prospective isolation of cells with different cycling rates • Does not require cloning or transgenic animals	• Limited dynamic range • Limited sensitivity for cells that are too homogeneous • Moderate cytotoxicity • Multiple measurements requiring 2 rounds of single-cell dissociation
Genetic label retention assay	Identification of nondividing cells based on retention of a stable fluorescent protein after expression is turned off	• Positively identifies nondividing label retaining cells, including rare populations *in vivo* that can be otherwise difficult to mark • Used in live animals	• Dividing cells don’t form well-resolved peaks, limiting sensitivity and dynamic range • Not a continuous monitor of cell cycle dynamics
H2B-FT reporter	Ratiometric reporter of cell cycle speed	• Single snapshot • Single-cell resolution • Enables FACS-based prospective isolation of cells with different cycling rates • Useful in live cells and animals • Continuously responsive to changes in cell division rate • Allows quantification of cell cycle rate for fast-cycling cells (<30h per cellcycle)	• Resolution among slow-cycling cells limited (only resolvable up to approximately 100h per cell cycle) • Occupies two fluorescent channels • Intensity must be captured in fresh tissue prior to fixation (blue form of FT is converted to red upon exposure to fixative) • Blue FT is photo-convertible, so z-stack imaging not enabled

a With caveats described in main text.

b Depending on model organism and whether the tissue is accessible for intravital microscopy.

## Abbreviations

AI: artificial intelligence
BrdU: Bromodeoxyuridine/5-bromo-2’-deoxyuridine
EdU: 5-ethynyl-2’-deoxyuridine
FACS: fluorescence activated cell sorting
FT: fluorescent timer
FUCCI: Fluorescent Ubiquitination-based Cell Cycle Indicator
H2B: histone H2B
IVF: *in vitro* fertilization
MFI: median fluorescent intensity
pH3: Phosphorylated Histone H3
scRNA-seq: single-cell mRNA sequencing
